# Cognitive interventions in mature and older adults, benefits for psychological well-being and quality of life: a systematic review study

**DOI:** 10.1590/1980-57642021dn15-040002

**Published:** 2021

**Authors:** Thais Bento Lima da Silva, Gabriela dos Santos, Ana Paula Bagli Moreira, Graciela Akina Ishibashi, Cássia Elisa Rossetto Verga, Luiz Carlos de Moraes, Patrícia Prata Lessa, Neide Pereira Cardoso, Tiago Nascimento Ordonez, Sonia Maria Dozzi Brucki

**Affiliations:** 1 Gerontology, School of Arts, Sciences, and Humanities, Universidade de São Paulo – São Paulo, SP, Brazil.; 2 Group of Cognitive and Behavioral Neurology, Hospital das Clínicas, Faculdade de Medicina, Universidade de São Paulo – São Paulo, SP, Brazil.; 3 Cognitive Training Study Group, School of Arts, Science and Humanities, Universidade de São Paulo – São Paulo, SP, Brazil.; 4 Instituto Supera de Educação – São José dos Campos, SP, Brazil.; 5 Department of Neurology, School of Medicine, Universidade de São Paulo – São Paulo, SP, Brazil.

**Keywords:** aging, educational activities, cognitive aging, social neuroscience, quality of life, depressive symptoms, envelhecimento, atividades educativas, envelhecimento cognitivo, neurociência social, qualidade de vida, sintomas depressivos

## Abstract

Few recently published studies investigating the benefits of educational and cognitive interventions on quality of life (QoL), psychological well-being, and depressive symptoms are available.

**Objective:**

The aim of this study was to investigate the effects of educational and cognitive interventions on psychological well-being, QoL, and mood in mature and older adults without dementia and/or with mild cognitive impairment (MCI).

**Methods:**

The systematic review took place from September to October 2020 and the following databases were used to select the studies: SciELO, LILACS, PubMed, and Medline. The search terms used were idos* AND “treino cognitivo” AND “bem-estar psicológico” AND “qualidade de vida” and their corresponding translations in English and Spanish.

**Results:**

Of the 241 articles retrieved, 26 primary studies were included in the review. Of these, 18 showed improvement in QoL, psychological well-being, or cognition.

**Conclusions:**

The studies reported beneficial effects of educational and cognitive interventions for QoL, psychological well-being, and depressive symptoms of mature and older adults without dementia or depression.

## RESUMO

Nos últimos anos, foram encontrados poucos estudos relatando os benefícios das intervenções cognitivas e educativas à qualidade de vida, ao bem-estar psicológico e contra os sintomas depressivos.

**Objetivo:**

Investigar os impactos de intervenções educativas e cognitivas nas variáveis de bem-estar psicológico, qualidade de vida e humor de adultos maduros e idosos sem demência e/ou com comprometimento cognitivo leve.

**Métodos:**

A pesquisa de revisão sistemática ocorreu entre setembro e outubro de 2020 e, para a seleção dos estudos, foram utilizadas as bases de dados: Scientific Electronic Library Online (SciELO), Literatura Latino-Americana e do Caribe em Ciências da Saúde (Lilacs), PubMed e Medical Literature Analysis and Retrieval System Online (MEDLINE). Foram selecionados os seguintes termos para a busca: “idos*” AND “treino cognitivo” AND “bem-estar psicológico” AND “qualidade de vida” e seus correspondentes em inglês e espanhol.

**Resultados:**

De 241 artigos contendo as palavras-chave, 26 estudos primários foram selecionados para esta revisão. Deles, 18 demonstraram melhora significativa na qualidade de vida, no bem-estar psicológico ou na cognição.

**Conclusões:**

Os estudos analisados evidenciaram efeitos positivos das intervenções educacionais e cognitivas para a qualidade de vida, para o bem-estar psicológico e contra os sintomas depressivos de idosos e adultos maduros sem demência e depressão.

## INTRODUCTION

Aging is a process associated with a host of biological, social, and psychological changes, including those affecting the cognitive domains. One of the main changes seen in cognitive ageing relates to executive functions, which support task planning and execution. Although there is a natural decline in these functions with age, the extent of this impairment is influenced by an individual’s extrinsic (environmental) and intrinsic (physical and mental) situation. Physical and cognitive abilities, that is, inherent characteristics, dictate functional capacity, promoting autonomous and independent living. This capacity has a significant impact on the quality of life (QoL) and well-being of older adults.^1,2,3^

The decline in functional capacity negatively impacts the QoL, defined as “an individual’s perception of his/her position in life in the context of the culture and value systems in which he/she lives, and about his/her goals, expectations, standards, and concerns.”^4^ Effects on psychological and social variables can manifest in the form of depressive symptoms, which are closely associated with general, subjective, and psychological well-being since depression undermines an individual’s sense of worth.^5^ The concept of subjective well-being encompasses emotional factors, positive or negative, and cognitive factors, more associated with personal satisfaction.^6^

Psychological well-being is related to six different factors: (1) autonomy: decision-making capacity; (2) purpose in life: having personal goals; (3) personal growth: developing new skills; (4) environmental mastery: management of situations according to context; (5) positive relations: maintaining ties with others; and (6) self-acceptance: having self-knowledge to accept oneself.^5,7^ One way of promoting maintenance of psychological well-being is through cognitive intervention.

Cognitive interventions can be divided into three types: (1) Cognitive Stimulation, in which standardized repeated activities are performed without structured learning programs; (2) Cognitive Training, in which standardized activities are established, targeting specific cognitive functions, such as attention, planning, reasoning, among others, while employing strategies to improve intervention outcomes; and (3) Cognitive Rehabilitation, aimed at recovery or compensatory strategies for cognitive impairments.^8,9^ In this study, our focus is on cognitive training and cognitive stimulation.

Cognitive training can positively influence psychological well-being and QoL, as found in the study by Irigaray et al.^10^ Their results showed that in addition to improvements in cognitive functions such as memory, language apraxias, there was a better perception of QoL and psychological well-being, with regard to the areas of self-acceptance, personal growth, and relationship with the environment. The authors stated that one reason that explains the effect of cognitive training on improving psychological well-being is that cognitive functioning in older adults is also influenced by psychological well-being. These results may indicate a positive association between improvements in cognition and psychological variables.

The study by Seinfeld et al.^11^ found that other types of intervention, such as piano practice and stimulation for cognitive domains — motor skills, attention, and executive function — also had beneficial effects in reducing depressive symptoms and improving mood in older adults. Thus, based on the results found, the authors suggested that playing piano at more advanced ages can help increase cognitive reserve, improving or maintaining cognitive function in later life — consistent with the findings of the cited studies. Therefore, although the study did not involve cognitive training per se, the results of the investigations were significant, suggesting that different types of cognitive interventions can improve the aspects of psychological well-being and QoL.

Among the actions used for health promotion, there are educational interventions, whose concept has not been much explored in the literature. What comes closest is the characterization described in the study by Carvalho et al.,^12^ who proposed that educational interventions are actions that would facilitate the understanding of the subjects involved, promoting the construction of new spaces of knowledge, through educational and dialogical relationships. Given the various intervention strategies presented, the integration of nursing in behavioral science also serves as a guide to explore the motivation or demotivation of the elderly, in addition to self-care in healthy aging, through the use of educational sessions with playful strategies in Social — Poetry workshops. Lima-Silva et al.^13^ mentioned that cognitive interventions added to cognitive stimuli can generate benefits for individuals’ self-efficacy, which is related to psychological well-being.

The absence of literature reviews on the effects of cognitive and educational interventions on subjective well-being, mood, and QoL of mature and elderly adults limits the possibility of gathering evidence that confirms or contradicts the results found in this study. However, clinical studies of cognitive training and cognitive stimulation have directed their results toward gains in terms of QoL and reduction of depressive symptoms,^14,5,16^ while studies of educational intervention have identified improvements in subjective well-being.^17,18^

In face of the explicit sociodemographic changes that take place in the course of the human existential trajectory, there is an increase in diseases involving mental health, particularly in the elderly population.^19^ However, there is scant literature about cognitive training in cognitively healthy older adults. There is a difficulty in finding studies in the literature that directly focus on the effects of cognitive training on QoL, psychological well-being, and depressive symptoms. This study did not identify other systematic reviews that aimed to ascertain the relationship between cognitive and educational interventions and psychological well-being, QoL, and mood. The only reviews that aimed to investigate the general gains of cognitive training were found.

Most studies found in the literature sample elderly people with preserved cognition, and associate cognitive training with improvements in memory, and also with improvements in social and psychological variables. However, there is a scarcity of comparative studies between the benefits of memory training for elderly people with MCI and for elderly people without any cognitive impairment, which limits the finding of which of the cognitive profiles benefit the most.

Therefore, considering the relevance of studies on cognitive and educational interventions and the gap of systematic reviews on the theme, this systematic literature review sought to address the importance of promoting and reporting the effectiveness of cognitive interventions, such as cognitive training and cognitive stimulation and educational interventions for the elderly, along with the benefits they can bring.

This study aimed to investigate the effects of educational and cognitive interventions on psychological well-being, QoL, and mood in mature and older adults without dementia and/or with MCI.

## METHODS

In this study, a systematic review of the literature was conducted according to the guidelines set forth in the PRISMA (Preferred Reporting Items for Systematic Reviews and Meta-Analyses) statement.^20^ The search and review of studies, carried out from September to October 2020, was performed on the SciELO, LILACS, PubMed, and Medline databases.

Using the PICo (Population, Interest and Context) framework,^20^ the following research question was defined: Can educational and cognitive interventions promote improvement in psychological well-being, mood, and QoL of older adults with MCI and those with depressive symptoms? The population refers to the mature and older adults without dementia and/or with MCI, the interest refers to the psychological well-being and QoL, and context refers to educational and cognitive intervention, including memory training and cognitive stimulation. After this process, eligible descriptors were selected on the Descriptors in Health Sciences (DeCS) system to serve as keywords for the search. The following search terms were employed: idos* AND “*treino cognitivo*” *AND* “*bem-estar psicológico*” *AND* “*qualidade de vida*,” their corresponding translations in English: elderly AND “*cognitive training*” *AND* “*psychological well-being*” *AND* “*quality of life*” *and Spanish: ancianos y* “*entrenamiento cognitivo*” *y* “*bienestar psicológico*” *and* “*calidad de vida.*”

The following filters were used to search for articles in the databases: “year 2010 to 2020,” “full text,” “randomized controlled trial,” and language “Portuguese,” “English,” and “Spanish.” Inclusion criteria were applied to articles published between 2010 and 2020. A process of inclusion and exclusion was carried out for the studies retrieved from each database congruent with this review. Inclusion criteria were: randomized clinically controlled trials, cohort studies, and studies involving older adults that were healthy, had MCI or comorbidities, and without neurodegenerative diseases. In addition, the studies should have been published in Portuguese, English, or Spanish. We included studies analyzing the effects on QoL or psychological well-being.

Exclusion criteria were secondary studies such as systematic reviews, meta-analyses, and studies conducted before 2010 and those that included older adults diagnosed with major neurocognitive disorders. Publications, which were case studies, master’s dissertations, doctoral theses, and book chapters, were also excluded. Other studies that failed to satisfy the predefined search criteria were also not included.

First, the titles and abstracts were read. Second, those who met the inclusion criteria, had their objectives, methods, and results filled out in a spreadsheet together with the name of the authors, the year of publication, and with the database website for a double check. The following data were observed: if the participants were 50 years or older; the application and effectiveness of the educational or cognitive intervention and; the use of instruments to assess the variables of subjective well-being and QoL in the pre- and posttest. Up to that stage, the research had been conducted by four reviewers. Finally, after methods, objectives, and results were checked, the selected articles were read completely by two researchers.

## RESULTS

**Figure 1.**
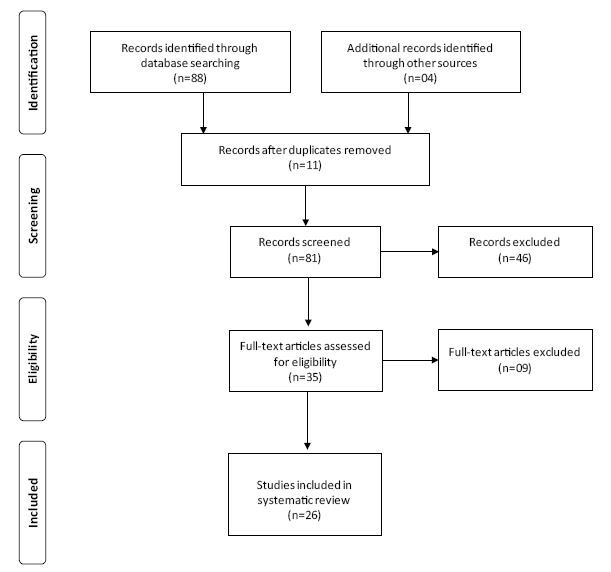
Flowchart of study search and review process.

The database searches led to the retrieval of 241 articles (6 PubMed, 3 Lilacs, 199 Medline, and 33 SciELO). After initial filtering for the inclusion of studies published between 2010 and 2020 plus RCTs and exclusion of duplicate studies, 81 articles remained, 4 of which were identified from paper references. Subsequent reading of titles and abstracts of the pre-selected articles led to the exclusion of a further 46, giving a total of 35 publications for a full reading. Finally, 26 studies met all inclusion criteria and were included as a part of this systematic review (Figure 1).

Of the 26 studies reviewed, most were published in 2018 (19.2%), 15 (57.7%) involved cognitive interventions, and the mean sample size was 212 participants. Concerning participants’ cognitive status, 21 (80.8%) studies involved cognitively healthy elderly, 3 (11.5%) investigated older people with and without MCI (11.5%), and 2 (7.7%) assessed groups with and without memory complaints. Published preliminary studies outlining methods only with no results were also assessed (Table 1).

**Table 1.** Summary of studies included.

Author/year	Objectives	Sample	Type of intervention	Results	Sample characteristics	Application effects
Birte-Antina et al. (2018)^22^	To investigate whether “olfactory training” (OT) had positive effects on subjective well-being and cognitive function.	n=91	Cognitive (training)	Analyses showed significant improvement in olfactory function for the OT group participants and improved verbal function and subjective well-being. Also, results indicated a decrease in depressive symptoms.	Cognitively healthy	Quality of life (QoL), mood, psychological well-being.
Vaportzis et al. (2017)^23^	To test the efficacy of a tablet computer training intervention to improve cognitive abilities of older adults.	n=48	Cognitive (stimulation)	A 2×2 mixed-model ANOVA suggested the tablet intervention group (n=22) had more significant improvements in Processing Speed (η^2^=0.10) compared with controls (n=21), but no difference in Verbal Comprehension, Perceptual Processing, or Working Memory (WM) (η^2^ range=0.03–0.04).	Cognitively healthy	QoL
Seinfeld et al. (2013)^11^	To study the specific effects of musical training vs. effects of other leisure activities in older adults. The impact of piano training on cognitive functioning, mood, and QoL was evaluated in older adults.	n=29	Cognitive (WM training)	The piano training group (TG) on the Stroop test measured executive function, inhibitory control, and divided attention in the piano TG. A tendency indicating enhancement of visual scanning and motor ability was also found (Trail-Making Test part A). Piano lessons decreased depression, induced positive mood states, and improved the psychological and physical QoL of the elderly. Results suggested that playing piano and learning to read music can help older adults promote cognitive reserve (CR) and improve subjective well-being.	Cognitively healthy	Mood, QoL
Tse et al. (2012)^27^	To examine the effects of an 8-week integrated pain management program (IPMP) on enhancing the knowledge and attitude toward pain management among staff; and improving pain, QoL, physical and psychosocial functions, and use of non-drug therapies for the elderly in nursing homes.	n=535	Cognitive (stimulation)	The staff showed significant improvement in knowledge and attitude to pain management, with the survey score increasing from 8.46±3.74 to 19.43±4.07 (p<0.001). Among residents, 74% had experienced pain within the previous 6 months, with a pain intensity of 4.10±2.20. Those in the experimental group (EG) showed a more significant reduction in pain scores than the control group (CG), from 4.19±2.25 to 2.67±2.08 (p<0.001). Group differences were also found in psychological well-being, including happiness, loneliness, life satisfaction, and depression (p<0.05) and in the use of non-drug methods (p<0.05).	Cognitively healthy	Psychological well-being
Gates et al. (2011)^24^	To report the rationale and methodology of the first trial to investigate isolated and combined effects of cognitive training (CT) and progressive resistance training (PRT) on general cognitive functioning and functional independence in older adults with early cognitive impairment in the Study of Mental and Regular Training (SMART). The secondary aim was to quantify differential adaptations to these interventions in terms of brain morphology and function, cardiovascular and metabolic function, exercise capacity, psychological state, and body composition, to identify the potential mechanisms of benefit and broader health status effects.	n=132	Cognitive (computerized CT)	Required sample size 10% larger than expected effect size (ES) (n=132) was originally estimated. The retention rate was >90%, confirming an appropriate recruitment target of 120/0.90=133. Thus, 80/133 were recruited, 60% of the planned cohort. Compliance with training sessions was high for all groups, with a median ranging from 78.44% for sham physical/CT to 100% for PRT/CT. One adverse event was reported (rotator cuff injury managed conservatively) in the PRT group, and no adverse events during assessments, CT, or sham interventions.	Cognitively healthy	No results
Maria Netto et al. (2012)^14^	To investigate a memory rehabilitation program’s therapeutic effect with assessments pre- and post-intervention in a group of older adults with mnemonic complaints and depressive symptoms.	n=7	Cognitive (stimulation)	Cognitive performance pre- and post-intervention was compared using the Wilcoxon test. There was a reduction in memory complaints and depressive symptoms, increased attentional processing speed, and improved WM. The authors suggested replicating the study in larger samples and groups with objective memory impairments and clinically diagnosed depression vs. CGs.	Cognitively healthy (with memory complaints)	Mood
Schultheisz et al. (2018)^29^	To determine the effects of a cognitive stimulation program on self-esteem and cognition of older persons.	n=38	Cognitive (stimulation)	Results showed that training improved performance on the cognitive test by older adults with and without cognitive impairment. This improvement had a subsequent positive effect on self-esteem.	Cognitively healthy (20 elderly people without cognitive complaints and 18 with cognitive complaints)	QoL
Chariglione et al. (2020)^15^	To determine whether cognitive gains promoted by two cognitive intervention programs were associated with improvements in mood, QoL, physical fitness (maximum oxygen consumption, lean mass, fat percentage, and handgrip strength) in older adults.	n=39	Cognitive (stimulation)	Both groups showed a tendency for reduced depressive symptoms, increased fat mass, and decreased lean mass.	Without and with cognitive decline	Mood
Ordonez et al. (2017)^16^	To investigate the effects of an electronic game program (Actively Station) on global cognitive performance in adults >50 years.	n=124	Cognitive (stimulation)	TG’s cognitive performance improved significantly after the program, particularly in language and memory domains, and there was a decrease in anxiety index and frequency of memory complaints compared to CG.	Cognitively healthy	Mood
Silva et al. (2011)^28^	To test the efficacy of a CT program based on ecological tasks that simulated shopping tasks, involving memorizing grocery items and performing simple mathematical calculations.	n=12	Cognitive (training – categorization)	The TG showed significant improvements, particularly on verbal fluency test – animals category and on immediate recall of words list (sum of three trials) of CERAD battery. No significant difference was found for the CG in pre- and post-test performance for variables assessed. Results suggested CT can increase performance in memorizing and calculation tasks among elderly adults.	Cognitively healthy	Psychological well-being
Chariglione and Janczura (2013)^30^	To investigate the influence of different CT procedures in memory, neuropsychological measures, and mood of institutionalized elderly and examine the relationship between educational level and intervention results. The study also sought to provide health professionals with a tool for aiding the rehabilitation of institutionalized elderly.	n=16	Cognitive (training)	Results showed the ARFC was influenced by training type and that free recall of words improved after training only for literate individuals, regardless of training type. No positive effects were detected in picture recognition, whereas training sessions positively influenced GDS scores.	Cognitively healthy	Mood
Lopes and Argimon (2016)^31^	To characterize the elderly participants and measure the effects of CT, with emphasis on executive functions, vs. a CG.	n=83	Cognitive (training)	Results showed significant group differences for the number of errors on the Sternberg Paradigm and Completed Categories of the WCST and Symbol Search.Intragroup comparisons showed EG had substantial improvement in scores post-intervention on GDS, RAVLT, Rey Complex Figures – memory, Forward Digit Span, and Total Digit Span Vocabulary tasks.	Cognitively healthy	Mood
Hagovská et al. (2017)^25^	To compare the effectiveness of two types of CT in 60 older adults with MCI by assessing the impact on functional activities, QoL, and various cognitive functions. The primary outcomes were functional activity level and QoL.	n=60	Cognitive (training)	After training, group A had better QoL (p<0.001, ES=0.69) and attention (increased loading score, p<0.05, ES=-0.23; errors, p<0.001, ES=-0.47); however, there were no group differences in functional activity level. Group A demonstrated greater improvements in QoL and attention than group B (i.e., classical CT), but the transfer to functional activities was same for both groups.	MCI	QoL, psychological well-being.
Gross et al. (2018)^32^	Structural equation modeling (SEM) was used to model hypothesized and novel relationships between constructs using available measures in ACTIVE based on the ACTIVE conceptual framework (Jobe et al., 2001; Figure 1). Each construct was first separately factor-analyzed in a measurement model to optimize fit to the data. Measurement models were then combined for all constructs together in a single model to explore hypothesized and additional pathways. In an extension to the ACTIVE conceptual framework, effect modification by demographic and health variables was tested. It was hypothesized that the data would fit the framework well, but *a priori* hypotheses about effect modifiers were not specified.	n=2802	Cognitive (training)	Preconceived measurement models for memory, reasoning, processing speed, everyday problem-solving, instrumental activities of daily living (IADL) difficulty, everyday speed, driving difficulty, and health-related QoL each fit well to the data (RMSEA<0.05; CFI>0.95). Fit of full model was excellent (RMSEA=0.038; CFI=0.924). In contrast with previous ACTIVE findings regarding who benefits from training, interaction testing revealed associations between proximal abilities and primary outcomes were generally stronger among individuals of non-white race, with worse health, older age, and less education (p<0.005).	Cognitively healthy	QoL
Buitenweg et al. (2019)^26^	To assess whether 12 weeks of cognitive flexibility training leads to improvement in subjective cognitive failures and executive dysfunctioning, everyday functioning, depressive symptoms, anxiety, and QoL.	n=158	Cognitive (training)	Subjective cognitive failures and executive dysfunctioning improved four weeks post-training in all groups, although ESs were small (ηp^2^=0.058 and 0.079, respectively), and there were no differences between groups (all p’s>0.38). No significant changes in subjective reports were seen directly after training, which was the case in all groups. Proxies reported no functional changes over time, yet their evaluations were significantly more favorable than those of the participants, both pre-training (p<0.0005) and post-training (p=0.004).	Cognitively healthy	QoL
Lucertini et al. (2019)^33^	The “TRIPL-A” study (i.e., a TRIal to promote Physical Activity among patients in the young-old affected by T2D) aimed to assess whether performing an innovative Exercise Referral Scheme (ERS), based on close collaboration among general practitioners, specialist physicians, exercise specialists and patients, supported by a web-based application (WBA), can effectively lead sedentary older T2D patients to adopt an active lifestyle.	n=300	Educational	Primary and secondary outcome results will evaluate the effectiveness of an ERS, specifically designed for the management of T2D clinical conditions and supported by a WBA, in promoting physical activity within Italian primary care settings.	Cognitively healthy	No results
Gonzalez-Hernandez et al. (2018)^34^	To study the efficacy of the CBCT protocol in a breast cancer survivor sample on QoL, psychological well-being, fear of cancer recurrence (FCR), self-compassion, and compassion domains and mindfulness facets. Enrolment, adherence, and satisfaction with the intervention were also analyzed.	n=56	Educational	The Accrual of eligible participants was high (77%), and the drop-out rate was 16%. Attendance of CBCT sessions was high, and practice outside sessions exceeded expectations. CBCT effectively diminishes stress caused by FCR, fostering self-kindness and common humanity, and increasing overall self-compassion scores, mindful observation, and acting with awareness skillsets.	Cognitively healthy	QoL, mood, psychological well-being.
Cantarella et al. (2017)^17^	To evaluate the effectiveness of a six-session Psychological Well-Being (PWB) intervention to improve PWB and identify transfer effects on an aspect related to PWB, QoL. Transfer effects on a high-level cognitive process, WM, were also investigated.	n=32	Educational	Only the trained group reported more significant gains in PWB and WM performance after training.	Cognitively healthy	Psychological well-being.
Thomson and Chatterjee (2014)^36^	To determine whether therapeutic benefits could be measured objectively using clinical scales.	n=40	Educational (ludic)	Positive affect and wellness increased significantly in acute and elderly and residential care though not psychiatric care, whereas negative affect decreased and happiness increased in all settings. Examination of audio recordings revealed enhanced confidence, social interaction, and learning. The program allowed adults access to a museum activity that would not otherwise have engaged with museum objects by age and ill-health.	Cognitively healthy (part of the group had anxiety and depression, but the results were described separately).	Mood, psychological well-being
Sales et al. (2015)^35^	To evaluate the effectiveness of an exercise intervention using an exercise park specifically designed for older people in reducing the risk of falls.	n=120	Educational	The BOOMER balance test was to be used as the primary outcome measure. Secondary outcome measures included handgrip strength, 2-minute walk test, lower limb strength test, spatiotemporal walking parameters, health-related QoL, feasibility, adherence, safety, and several other psychosocial measures. Outcome assessments were to be conducted at baseline and 18 and 26 weeks after intervention commencement. Participants would report falls and physical activity history for 12 months via monthly calendars. Mixed linear modeling incorporating intervention and CGs at baseline and two follow-ups (18 weeks and 26 weeks after intervention commencement) would be used to assess outcomes.	Cognitively healthy	No results
Mavrovouniotis et al. (2010)^18^	To examine the effect of Greek traditional dances on the improvement of older people’s QoL.	n=111	Educational (ludic)	Independent-group t tests showed that the CG, compared to the EG, at rest and on the second measurement, had significantly higher levels of state anxiety, psychological distress, fatigue, and significantly lower levels of positive well-being. After dancing, approximately 63% of the maximum heart rate was achieved in the EG. At the same time, paired t tests revealed significant decreases in state anxiety and psychological distress, as well as substantial increases in positive well-being and fatigue.	Cognitively healthy	Psychological well-being.
Lima-Silva et al. (2010)^13^	To test the efficacy of a CT program based on the creation of mental images and changes in specific aspects of meta-memory in individuals with 3–15 years of education (M=8.38, SD=4.24).	n=32	Cognitive (training) and Educational	The TG showed significant improvement between pre- and post-tests on delayed recall of 10 pictures and self-efficacy to memorize stories. These same changes were not found in the CG.	Cognitively healthy	Mood
Irigaray et al. (2011)^10^	To investigate the effects of a CT program on the QoL and psychological well-being of healthy elderly.	n=76	Cognitive (training) and Educational	Results showed that healthy elderly could benefit from this type of intervention, reducing conditions that lead to pathological cognitive aging and promoting QoL and psychological well-being in old age.	Cognitively healthy	QoL, psychological well-being
Irigaray et al. (2012)^38^	To investigate the effects of an attention, memory, and executive functions training intervention on healthy older adults’ cognition.	n=76	Cognitive (training) and Educational	Post-test, the EG had better performance on attention, WM, language (inferences and spontaneous writing), constructional praxis, problem-solving, and executive function tasks. The training produced significant results after a 12-session intervention, indicating that healthy elderly individuals’ cognitive functioning can be improved.	Cognitively healthy	Mood
Chandler et al. (2019)^39^	To compare the cumulative effects of combinations of five behavioral interventions on significant outcomes in patients with MCI.	n=272	Cognitive (stimulation) and Educational	272 participants (mean [SD] age, 75 [8] years; 160 [58.8%] males and 112 [41.2%] females) were enrolled, with 56 randomized to no yoga group, 54 to no computerized CT, 52 to no wellness, 53 to no support, and 57 to no memory support system. The most remarkable ES for QoL was between no computerized CT and no wellness education groups (ES 0.34, 95%CI 0.05–0.64). On secondary analyses, wellness education had a greater effect than computerized CT on mood (ES 0.53; 95%CI 0.21–0.86), and yoga had a more significant impact than support groups on memory-related activities of daily living (ES 0.43; 95%CI 0.13–0.72).	Cognitively healthy and MCI	Psychological well-being.
Pérez et al. (2015)^40^	To assess the effectiveness of memory training workshops for healthy older people in terms of short- and long-term impact on cognitive function, health-related QoL, and functioning.	n=230	Cognitive (training) and Educational	Study results will be useful for social and public health policies related to older people. Given the increase in the prevalence of older people, many interventions targeting memory loss are funded by public resources. To ensure transparency and effective prioritization, such research is needed to provide evidence of these interventions’ effectiveness and usefulness.	Cognitively healthy	No results

ACTIVE: Advanced Cognitive Training for Independent and Vital Elderly, ANOVA: analysis of variance, ARFC: Brief Cognitive Function Assessment, BOOMER: Balance Outcome Measure for Elder Rehabilitation, CBCT: Cognitively Based Compassion Training, CERAD: Consortium to Establish a Registry for Alzheimer’s Disease, CFI: Comparative Fit Index, GDS: Geriatric Depression Scale, MCI: mild cognitive impairment, RAVLT: Rey Auditory–Verbal Learning Test, RMSEA: Root Mean Square Error of Approximation, T2D: type 2 diabetes, WCST: Wisconsin Card Sorting Test.

Many studies have found an association between reducing depression symptoms or increasing the QoL or psychological well-being of participants after the intervention. Among the studies, those which offered only educational intervention were the ones that identified an increase in psychosocial well-being. Regarding mood, those which carried out cognitive intervention such as cognitive stimulation, showed more results in reducing depressive symptoms, compared to studies with other types of intervention.

## DISCUSSION

This systematic review study aimed to analyze educational and cognitive interventions and determine their effects on associated subjective variables, such as QoL, psychological well-being, and depressive symptoms in cognitively healthy mature and older adults, subjects with MCI and no depression. The approach entailed analysis of several instruments and aspects associated with definitions of QoL and psychological well-being.^7,21^

Cognitive type interventions employ a range of different approaches: olfactory training,^22^ cognitive training using electronic devices, such as computers or tablets,^13,23,24,25,26^ training using musical instruments,^10^ training through manual interventions,^27^ training using categorization strategies,^28^ and multifactorial training,^14,15,25,29,30,31,32^ which proved the most predominant approach (7/15 cognitive intervention studies).

The majority of the studies reviewed, which involved cognitive nature activities, showed significant results for at least one of the variables assessed (QoL, psychological well-being, depressive symptoms, or mood). The study by Birte-Antina,^22^ whose sample received olfactory training, found improvements for cognition, psychological well-being, QoL, and depressive symptoms. By contrast, three studies failed to achieve significant results for QoL or psychological well-being post-intervention.^15,16,26^ Buitenweg et al.^26^ concluded that a possible explanation for nonsignificant effects in the sample investigated might lie in the fact that the elderly participants had high cognitive performance and low depression symptoms preintervention.

Educational type interventions tend to be used for promoting health, given that the individual is empowered to practice self-care and thus has greater adherence to professional practices.^12^ Studies with an educational approach in the present literature review focused on the following areas: healthy lifestyle,^33^ personal growth,^17,34^ falls prevention,^35^ and play-oriented activities involving museum object handling^36^ or dance.^18^

The study by Cantarella et al.,^17^ in which a group of women participated in classes and discussions aimed at favoring personal growth, found significant results for participants’ psychological well-being following the intervention. The results were consistent with the findings of the studies by Cachioni et al.^7^ and Ordonez et al.,^37^ assessing subjective and psychological well-being of elderly participants of an open Universidade da Terceira Idade, which identified positive effects of education on general satisfaction with life, where students engaged in the interventions for longer had more significant results.

Other studies combined educational interventions with cognitive interventions, incorporating discussions about memory, aging, and well-being in the cognitive training programs.^11,13,38,39,40^ The study by Irigaray et al.,^10^ investigating the effects of cognitive training on QoL and psychological well-being, found significant results for both variables. The researchers’ first hypothesis to explain this outcome involves the relationship between enhanced cognitive capacity and its benefits for QoL. At the same time, the secondary idea suggests a broadening of participants’ social circle.

However, one of the studies analyzed psychological well-being and depressive symptoms and failed to identify significant results.^5^ This outcome echoes Silva et al.,^28^ who also found no improvements in depressive symptoms in training or control groups after a cognitive training intervention in mature and older adults.

In summary, the studies reported positive effects of educational and cognitive interventions for QoL, psychological well-being, and depressive symptoms among mature and older adults without dementia or depression. These results suggest that different intervention strategies can benefit mature and older individuals, warranting further research on the topic.

The results corroborate another systematic review, which investigated the benefits of cognitive training, but without focusing on the three variables evaluated in this study. Gomes et al.,^41^ in an integrative review on the relationship between cognitive training and functionality in elderly people without cognitive impairment, found that 50% of the studies that were analyzed indicated positive effects of cognitive training on psychological, social, and QoL variables.

Santos and Mendoza,^8^ however, in a systematic review of national studies that involved cognitive training, warn of the need for caution when relating cognitive intervention to psychosocial gains. The authors claim that because these are variables measured through self-reporting, responses may be influenced by external factors, which compromises the validity of the information collected. In general, there is a lack of literature reviews on the theme brought up by this study, which prevents the comparison of the results found and, consequently, the application of greater scientific basis.

The main limitations of this review were a dearth of methodological processes in studies involving cognitive and educational interventions among cognitively healthy mature and older adults or those with MCI, particularly regarding the application of assessment instruments pre- and posttest. In addition, there was a lack of cognitive training interventions concomitantly investigating effects on QoL, psychological well-being, depressive symptoms, and mood among older adults. Another limitation was the absence of a calculation that could measure the effect size of the results found. In addition, there was a lack of prior registration of the review protocol in a database.

Despite the limitations, the results found sugg-est that there is a relationship between educational and cognitive interventions and psychological and QoL variables. Therefore, these practices can be applied by professionals who seek improvements beyond cognition and learning. This possibility allows the adoption of complementary strategies to provide psychosocial gains for mature and elderly adults.

It is suggested, for future research, that there is greater deepening in the comparison of the gains from educational interventions and the gains resulting from cognitive interventions.

## References

[1] Oliveira AS, Silva VC, Confort MF (2017). Benefícios da estimulação cognitiva aplicada ao envelhecimento. Rev Episteme Transversalis..

[2] Organização Mundial de Saúde (2015). Relatório mundial de envelhecimento e saúde.

[3] Karsch UM, Salimene AC, Oliveira B, Hayar MA (2019). Envelhecimento com dependência: cuidados e cuidadores de idosos.

[4] The World Health Organization quality of life assessment (WHOQOL) (1995). Position paper from the World Health Organization. Soc Sci Med..

[5] Oliveira DS de, Lima MP, Ratto CG, Rossi T, Baptista RR, Irigaray TQ (2020). Avaliação de bem-estar psicológico e sintomas depressivos em idosos saudáveis. Estud Pesqui Psicol..

[6] Teixeira CR, Scortegagna SA, Portella MR, Pasian SR (2019). Bem-estar subjetivo de longevos institucionalizados e não institucionalizados por meio do Pfister. Aval Psicol [serial on line].

[7] Cachioni M, Delfino LL, Yassuda MS, Batistoni SS, Melo RC, Domingues MA (2017). Subjective and psychological well-being among elderly participants of a University of the Third Age. Rev Bras Geriatr Gerontol..

[8] Santos MT, Flores-Mendoza C (2017). Treino cognitivo para idosos: uma revisão sistemática dos estudos nacionais. Psico-USF..

[9] Golino MT, Flores-Mendoza C (2016). Desenvolvimento de um programa de treino cognitivo para idosos. Rev Bras Geriatr Gerontol..

[10] Irigaray TQ, Schneider RH, Gomes I (2011). Effects of a cognitive training on the quality of life and well-being of healthy elders. Psicol Reflex Crit..

[11] Seinfeld S, Figueroa H, Ortiz-Gil J, Sanchez-Vives MV (2013). Effects of music learning and piano practice on cognitive function, mood and quality of life in older adults. Front Psychol..

[12] Carvalho KM, Silva CR, Figueiredo ML, Nogueira LT, Andrade EM (2018). Intervenções educativas para promoção da saúde do idoso: revisão integrativa. Acta Paul Enferm..

[13] Lima-Silva TB, Ordonez TN, Santos GD, Fabrício AT, Aramaki FO, Almeida EB (2010). Effects of cognitive training based on metamemory and mental images. Dement Neuropsychol..

[14] Maria T, Fonseca RP, Landeira-Fernandez J (2012). Reabilitação da memória em idosos com queixas mnemÃ´nicas e sintomas depressivos: estudo piloto não controlado. Estud Psicol (Natal)..

[15] Chariglione IP, Silva HS, Melo GF, Vilaça e Silva KH, Oliveira ML (2020). Cognitive interventions and performance measures: a longitudinal study in elderly women. Estud Psicol (Campinas)..

[16] Ordonez TN, Borges F, Kanashiro CS, Santos CC, das N, Hora SS, Lima-Silva TB (2017). Actively station: Effects on global cognition of mature adults and healthy elderly program using electronic games. Dement Neuropsychol..

[17] Cantarella A, Borella E, Marigo C, De Beni R (2017). Benefits of well-being training in healthy older adults. Appl Psychol Health Well Being..

[18] Mavrovouniotis FH, Argiriadou EA, Papaioannou CS (2010). Greek traditional dances and quality of old people’s life. J Bodyw Mov Ther..

[19] World Health Organization (2020). Dementia [serial on line].

[20] Fragoso YD, Campos NS, Tenrreiro BF, Guillen FJ (2012). Systematic review of the literature on vitamin A and memory. Dement Neuropsychol..

[21] Neri AL (2014). Palavras-chave em Gerontologia.

[22] Birte-Antina W, Ilona C, Antje H, Thomas H (2018). Olfactory training with older people. Int J Geriatr Psychiatry..

[23] Vaportzis E, Martin M, Grow AJ (2017). A tablet for healthy ageing: the effect of a tablet computer training intervention on cognitive abilities in older adults. Am J of Geriatr Psychiatry..

[24] Gates NJ, Valenzuela M, Sachdev PS, Singh NA, Baune BT, Brodaty H (2011). Study of Mental Activity and Regular Training (SMART) in at risk individuals: A randomised double blind, sham controlled, longitudinal trial. BMC Geriatr..

[25] Hagovská M, Dzvoník O, Olekszyová Z (2017). Comparison of two cognitive training programs with effects on functional activities and quality of life. Res Gerontol Nurs..

[26] Buitenweg JI, Ven RM, Ridderinkhof KR, Murre JM (2019). Does cognitive flexibility training enhance subjective mental functioning in healthy older adults?. Neuropsychol Dev Cogn B Aging Neuropsychol Cogn..

[27] Tse MMY, Sin Vong SK, Ho SSK (2012). The effectiveness of an integrated pain management program for older persons and staff in nursing homes. Arch Gerontol Geriatr..

[28] Silva TB, Oliveira AC, Paulo DL, Malagutti MP, Danzini VM, Yassuda MS (2011). Cognitive training for elderly adults based on categorization strategies and calculations similar to everyday tasks. Rev Bras Geriatr Gerontol..

[29] Schultheisz TS, Aquino RR, Alves AB, Radl AL, Serafim AP (2018). Effect of cognitive stimulation workshops on the self-esteem and cognition of the elderly A pilot project. Dement Neuropsychol..

[30] Chariglione IP, Janczura GA (2013). Contributions of a cognitive training for the memory of institutionalized elderly. Psico-USF..

[31] Lopes RM, Argimon II (2016). Cognitive Training in the elderly and its effect on the executive functions. Act Colom Psicol..

[32] Gross AL, Payne BR, Casanova R, Davoudzadeh P, Dzierzewski JM, Farias S (2018). The ACTIVE conceptual framework as a structural equation model. Exp Aging Res..

[33] Lucertini F, Ferri Marini C, Sisti D, Stocchi V, Federici A, Gregorio F (2019). Discontinuously supervised aerobic training vs. physical activity promotion in the self-management of type 2 diabetes in older Italian patients: design and methods of the ‘TRIPL-A’ randomized controlled trial. BMC Geriatr..

[34] Gonzalez-Hernandez E, Romero R, Campos D, Burychka D, Diego-Pedro R, Baños R (2018). Cognitively-Based Compassion Training (CBCT®) in breast cancer survivors: a randomized clinical trial study. Integr Cancer Ther..

[35] Sales MP, Polman R, Hill KD, Karaharju-Huisman T, Levinger P (2015). A novel dynamic exercise initiative for older people to improve health and well-being: study protocol for a randomised controlled trial. BMC Geriatr..

[36] Thomson LJ, Chatterjee HJ (2014). Well-being with objects: evaluating a museum object-handling intervention for older adults in health care settings. J Appl Gerontol..

[37] Ordonez TN, Lima-Silva TB, Cachioni M (2011). Subjective and psychological well-being of students of a University of the Third Age. Dement Neuropsychol..

[38] Irigaray TQ, Gomes I, Schneider RH (2012). Effects of an attention, memory and executive functions training on the cognition of healthy elderly people. Psicol Reflex Crit..

[39] Chandler MJ, Locke DE, Crook JE, Fields JA, Ball CT, Phatak VS (2019). Comparative effectiveness of behavioral interventions on quality of life for older adults with mild cognitive impairment: a randomized clinical trial. JAMA Netw Open..

[40] Pérez A, Roqué M, DomÃ¨nech S, Monteserín R, Soriano N, Blancafort X (2015). Efficacy of memory training in healthy community-dwelling older people: study protocol for a randomized controlled trial. BMC Geriatr.

[41] Gomes EC, Souza SL, Marques AP, Leal MC (2020). Memory stimulation training and the functionality of the elderly without cognitive impairment: an integrative review. Ciênc Saúde Coletiv..

